# Proline, glutamic acid, and leucine rich protein 1 (PELP1) expression in deep paravertebral muscles in idiopathic scoliosis girls

**DOI:** 10.1186/1748-7161-10-S1-O5

**Published:** 2015-01-19

**Authors:** Malgorzata Kotwicka, Izabela Skibinska, Miroslaw Andrusiewicz, Tomasz Kotwicki

**Affiliations:** 1 Department of Cell Biology, Poznan University of Medical Sciences, Poland; 2 Department of Pediatric Orthopedics and Traumatology, Poznan University of Medical Sciences, Poland

## Objectives

Estrogens and their receptors are postulated to be important factors in development of idiopathic scoliosis. Transcription functions of estrogen receptors are influenced by coregulators such as proline, glutamic acid, and leucine rich protein 1 (PELP1). Deep paravertebral muscles are potential elements participating in idiopathic scoliosis development. The aim of the present study was to evaluate PELP1 mRNA expression levels and the protein presence in deep paravertebral muscles on both sides of the spinal curve and correlation between the expression level and scoliosis parameters.

## Material and methods

The study comprises 29 girls with idiopathic scoliosis who underwent posterior spinal surgery. Average age was 15 years and 4 months. Radiological examination consisted of Cobb angle measurement and bone maturity evaluation based on Risser sign. Muscle sample were harvested from superficial (trapezius) and deep paravertebral (longissimus) muscles at both sides of the apical area. Presence of PELP1 gene transcripts was investigated using RT-qPCR technique. Immunohistochemistry and western blot methods were used to confirm PEPL1 protein presence.

## Results

Cobb angle of the main thoracic curve ranged from 52degree to 114degree. Progression risk factor ranged from 1.9 to 8.5. RT-qPCR revealed expression of PELP1 transcript in superficial and deep paravertebral muscles. Level of PELP1 expression in deep paravertebral muscles was significantly higher than in superficial muscles (p=0.005) and did not differ between convex and concave side (p>0.05), however in 6 patients with Cobb angle > 95degree a higher expression in paravertebral muscles on the concave side of the curve was observed (p<0.05). Positive immunohistochemical staining for PELP1 was detected in the nuclei of the back muscles cells. No cytoplasmic staining was observed. Western blot analysis, performed on tissue samples obtained from randomly chosen patients, revealed presence of PELP1 protein in all samples.

## Conclusion

Three independent techniques for the first time revealed presence of PELP1 protein in deep paravertebral muscles of patients with idiopathic scoliosis, taking into consideration its potential regulatory influence on back muscle function.

